# Supplementary material for: Collaborative intelligence in AI: Evaluating the performance of a council of AIs on the USMLE
Source: PLOS Digit Health. 2025 Oct 9;4(10):e0000787. doi: 10.1371/journal.pdig.0000787 (PMC12510544; doi:10.1371/journal.pdig.0000787)
Supplement: S2 File — (ZIP) [file pdig.0000787.s002.zip › S2 Data Raw data files containing unprocessed question inputs and LLM outputs including transcripts of deliberations/STEP3_Question-109-1_Type-MC-NJ.docx]

**Facilitator** **[Round 1]:** A 55-year-old woman is brought to the office by her husband because of increasingly severe pain in her mid back for the past week. She says the pain is especially severe with walking. Her husband says she has been favoring her right leg for the past 3 days. Ibuprofen no longer controls the pain. She enjoys gardening but had to discontinue it 1 week ago because of the pain. She has not had bowel or bladder dysfunction. She underwent a right mastectomy 3 years ago for carcinoma and has been taking tamoxifen since that time. She is 173 cm (5 ft 8 in) tall and weighs 66 kg (145 lb); BMI is 22 kg/m². Vital signs are temperature 38.0°C (100.4°F), pulse 90/min, respirations 15/min, and blood pressure 118/72 mm Hg. Strength is 3/5 in all muscle groups of the right lower extremity and 5/5 in the left lower extremity. Achilles and patellar reflexes are hyperactive on the right and normal on the left. Sensation to pinprick and temperature is decreased in the left lower extremity to the level of the inguinal ligament. Sensation to vibration is decreased in the right lower extremity. Reflexes and sensation are otherwise intact. Rectal sphincter tone is normal. Which of the following is the most appropriate next step? (A) Acetaminophen-oxycodone therapy and referral for physical therapy (B) Admission to the hospital for pain control (C) Electromyography and nerve conduction studies (D) MRI of the thoracic spine (E) Technetium 99m scan

**AI_A:** RESTATING THE QUESTION: In simpler terms, we have a 55-year-old woman who has serious back pain that gets worse with walking. She has been limping on her right leg. She has a history of breast cancer, and medications do not seem to help with her pain. She has no bowel or bladder issues. Her physical examination indicates some neurological abnormality as one leg is weaker than the other, reflexes are more pronounced on one side, and there is decreased sensation of different types in both legs. Despite the above, her rectal tone is normal. Where should we go from here to help this patient? PLANNING: My assumptions include the woman's back pain is associated with her cancer history and tamoxifen treatment, her lack of bowel or bladder dysfunction contradicts spinal cord compression, her unilateral weakness, hyperactive reflexes and decreased sensation indicate neurological involvement, her fever might indicate an infection or an inflammatory process, and her normal rectal sphincter tone contradicts a cauda equina syndrome. The main assumption I am hypothesizing and wish to prioritize is the possibility of cancer metastasis to the spine causing spinal cord compression, which can explain her mid back pain, unilateral weakness, hyperactive reflexes and decreased sensation. CLINICAL REASONING: Cancer metastasis to the spine as a result of her breast carcinoma could be the cause of her back pain. Pathologically, carcinomas, especially breast carcinomas, have a predilection to metastasize to the spine. This relates to the unique blood flow in the vertebral bodies that allows tumor cells to deposit there. A spine metastasis could directly compress the spinal cord or cause instability leading to movement-induced pain. Depending on location, it could cause weakness, hyperactive reflexes and sensation changes. The unilaterality of findings could be associated with lateral (one side) cord compression. Metastasis might also provoke inflammatory response causing fever. However, tamoxifen use as well can lead to pathophysiological changes like thrombosis, which could result in similar findings. CONCLUDING: Given her history of breast carcinoma, current symptoms and neurological examination findings, it is essential to rule out metastatic disease in the spine causing spinal cord compression. This can account for her back pain, neurological deficits and possibly her fever. An imaging study will be essential in this case. RESPONSE: The most appropriate next step is (D) MRI of the thoracic spine, since it allows us to visualize the spinal cord, any possible compressive lesion, and their relationship to the surrounding structures. Other options might be beneficial later, depending on the MRI findings.

**AI_C:** RESTATING THE QUESTION: This patient, a 55-year-old lady who has previously undergone a mastectomy for breast cancer, is experiencing worsening mid-back pain especially aggravated by walking, resulting in her right leg being favored as her pain is not relieved by ibuprofen. She demonstrates hyperactive right-side lower extremity reflexes, decreased strength in the same region, reduced sensation to pinprick, temperature in the left lower extremity and to vibration in the right. The possible next courses of action include numerous interventions such as acetaminophen-oxycodone and physical therapy, hospitalization for pain management, electromyography and nerve conduction studies, MRI of the thoracic spine, or a Technetium 99m scan. We are asked to determine the most appropriate next step. PLANNING: The assumptions under consideration here are that her symptoms could be related to her history of carcinoma (given the mastectomy and tamoxifen treatment), but could also be due to an unrelated condition. A further assumption might be that her symptoms are musculoskeletal or neurological in nature since she presents with mixed neural deficits. Imaging of the thoracic spine or a Technetium 99m scan could be clinically prioritized given the unclear etiology of her current condition, specifically the presence of differential sensory and motor neurological deficits. Imaging may reveal a central cause like a lesion impinging upon the spinal cord or nerves. CLINICAL REASONING: The woman demonstrated a history of cancer and her new symptoms may suggest metastatic invasion to her spinal cord presenting as severe back pain, reduced strength and sensory changes. The differential sensory loss (pinprick and temperature on the left, vibration on the right) is characteristic of a spinal cord lesion in what is known as a Brown-Sequard syndrome, which is often due to a unilateral lesion in the spinal cord. This could be due to a mass (like metastasis) impinging on the right side of her spinal cord. CONCLUDING: From the information at hand, the pattern of her neurological deficits is suggestive of a central cause rather than a peripheral one. An MRI of the thoracic spine is therefore more appropriate compared to electromyography or nerve conduction studies which primarily evaluate peripheral nerve disorders. The step-by-step clinical thought process indicates that this woman likely has central nerve pathology (potentially from metastasis), thus advancing testing with an MRI of the thoracic spine might be more beneficial. This would allow further visualization and bloke us to identify any potential lesions causing these signs and symptoms. RESPONSE: Therefore, given the patient's symptoms, history of carcinoma, and her neurologic signs consistent with a central lesion, (D) MRI of the thoracic spine is the most appropriate next step. It will provide the most valuable imaging of potential lesions, such as a spinal metastasis, causing her signs and symptoms.

**AI_E:** RESTATING THE QUESTION: The woman in this scenario is experiencing progressively worse mid-back pain which effects her mobility and does not respond to ibuprofen. Additionally, there are noted discrepancies in her strength, reflex, and sensory evaluations. Importantly, three years prior, she was treated for breast carcinoma through mastectomy and has been on tamoxifen therapy ever since. The question asks us to recommend the next appropriate course of action from a list of five options, which largely involve pain management, diagnostic evaluations, and imaging procedures. PLANNING: The important considerations in this case involve the woman’s back pain, changes in her right lower limb function and previous history of breast carcinoma. Given the severe and worsening nature of the pain, along with the differential function and sensation in the right leg and the fact that she had a breast carcinoma and has been on tamoxifen therapy, the underlying cause may be a metastatic lesion in her spinal column. Although non-steroidal pain medication and physical therapy (option A), hospital admission for pain control (option B), and nerve studies (option C) are valid interventions, they do not address the potential cause. Technetium 99m scan (option E) is a nuclear imaging test that could be used for various conditions but is less specific than MRI for spinal issues. An MRI of the thoracic spine (option D) provides a more detailed view of the spine regions, allowing detection of potential metastases or spinal cord compression, which may be causing the symptoms. CLINICAL REASONING: Given the history, physical findings, and response to pain relievers, the signs point towards an issue in the spinal region. The difference in strength, reflex and sensation, favoring of the right leg, and uncontrolled mid-back pain may be due to a lesion in the spinal canal, possibly metastasis from her previous breast carcinoma. MRI is the gold standard for visualizing spinal cord pathology. CONCLUDING: Taking all these factors into account, it would be reasonable to prioritize identifying the underlying issue, which may well be a spinal lesion causing the described symptoms, over immediate but potentially ineffectual pain management or non-specific imaging procedures. RESPONSE: So, considering the patient's back pain, right leg favoring, and history of breast cancer, the most appropriate next step would be: (D) MRI of the thoracic spine. This would allow for detecting any possible metastatic lesions or spinal cord compression that could be causing the symptoms.

**AI_B:** RESTATING THE QUESTION: The inquiry discusses a 55-year-old woman's medical case. For a week, she has experienced progressively worsening mid-back pain, more intense during walking. She has favored the right leg in the last 3 days, as observed by her husband. Despite the use of ibuprofen, the pain remains uncontrolled. She also enjoys gardening but had to stop due to the discomfort. No gastrointestinal or urinary abnormalities were noted. A history of right mastectomy due to carcinoma 3 years prior is given, where she has since then been prescribed tamoxifen. Her vital signs include a fever, and her neurologic exams show asymmetric findings. The question now points to the next most appropriate course of action, providing five options for consideration. PLANNING: Several assumptions could be made in this case. First, the severe mid-back pain could be connected to the patient's previous carcinoma diagnosis and use of Tamoxifen, which may suggest metastasis. Hyperactive reflexes and the decrease in strength and sensory function in different lower extremities suggest a possible spinal cord lesion. However, the assumption of metastasis should be clinically prioritized given the woman's oncologic history and occurrence of systemic symptoms such as fever. CLINICAL REASONING: Tamoxifen may lead to a higher risk for endometrial cancer but is otherwise instrumental in reducing the risk of breast cancer recurrence and mortality. This patient's described progressive, severe pain along her mid-back that escalates with walking could indicate the presence of a pathological process within her vertebral column – a red flag symptom. This is further supported by a diminished sensation to pinprick/temperature on one leg, reduced vibration sensation on the other, and hyperactive reflexes. Such distribution may be suggestive of a spinal cord lesion, possibly involving the sensory and motor tracts that supply the lower extremities. Given the patient's history of breast cancer, there is a concern that there might be metastasis to her spine. A spinal metastasis may present with back pain, which is worse with weight-bearing activities (e.g., walking), and neurological deficits, which aligns with this patient's clinical picture. CONCLUDING: Careful clinical considerations would point towards possibility of metastasis to the spine—aligning the woman's ongoing breast cancer treatment, her unaccounted fever, and the neurologic findings. To further validate the assumption, the next step would involve an imaging test that could potentially reveal possible lesions linked to metastasis in her spine. RESPONSE: Given the patient's symmetrical neurological findings suggesting a spinal cord lesion and her history of previous breast cancer, the most plausible next step in management at this time would be (D) an MRI of the thoracic spine to investigate the possibility of metastasis.

**AI_D:** (1) RESTATING THE QUESTION: A woman of 55 years who has a history of right mastectomy for carcinoma, now reports experiencing severe back pain for the past week. The pain is severe while walking and forces her to favor her right leg, making it difficult for her to continue with her hobbies such as gardening. Notably, painkillers such as ibuprofen no longer help to control the pain. There are no bowel or bladder dysfunctions reported. Physical examination reveals a decreased muscle strength (3/5) in all muscle groups of the right lower extremity and hyperactive Achilles and patellar reflexes on the right side. There is a decrease in sensation to pinprick, temperature, and vibration in the lower extremities. However, rectal sphincter tone is normal. The query revolves around what will be the best next course of action, from options like drug therapy, hospital admission, EMG and nerve conduction studies, MRI of the thoracic spine, or technetium 99m scan. (2) PLANNING: Considering the combination of clinical findings which include back pain, decreased strength, and abnormal sensations in the extremities, one plausible assumption could be that the woman might have a spinal lesion. The hyperactive reflexes could often signify a lesion in the upper motor neuron, while the decreased sensations could indicate a problem in the dorsal column-medial lemniscus pathway and the anterolateral system. These symptoms, combined with her past medical history of carcinoma, could raise the possibility of metastatic cancer to the spine. This could be a reasonable assumption to explore initially, while also considering alternate assumptions such as disc herniation, spinal abscess, or spinal stenosis. (3) CLINICAL REASONING: Metastatic cancer to the spine often presents with back pain, which may be severe enough to limit physical activities such as walking or gardening, as in this case. This can be associated with motor deficits (weakness) and sensory disturbances, as seen in this patient. The pain may not be relieved adequately by non-steroidal anti-inflammatory drugs such as ibuprofen. Thus, the symptoms correlate well with the hypothesized assumption of metastatic disease. (4) CONCLUDING: Given the suspected clinical diagnosis of metastasis to the spine, pathological confirmation and localization of the lesion would be crucial for management, which may include surgery, radiotherapy, or chemotherapy. Therefore, to verify this assumption, an imaging study that provides detailed views of the bony and soft tissues of the spine would be necessary. (5) RESPONSE: Considering her history, physical examination findings, and relevant clinical assumptions, the most appropriate next step would be (D) MRI of the thoracic spine. The MRI study would not only confirm the presence of a spinal lesion but could also provide insight into whether the lesion is extradural (common with metastasis), intradural-extramedullary, or intramedullary. Detailed views might show pathologies such as metastatic lesions or any associated spinal cord compression, which will then guide further interventions. While Technetium 99m scan could also detect metastasis, it is less specific and would not provide information about exact localization or cord compression. Therefore, MRI is the better choice for initial investigation.

**Facilitator** The consensus statement is as follows: The case involves a 55-year-old woman with a history of breast carcinoma, who is experiencing severe mid-back pain that worsens with walking, favoring of her right leg, and uncontrolled pain despite ibuprofen use. Her physical examination reveals neurological abnormalities, including decreased strength in the right lower extremity, hyperactive reflexes on the right side, and decreased sensation in both lower extremities. Given her history and symptoms, the primary assumption is that her condition may be related to metastasis of her previous breast carcinoma to the spine, potentially causing spinal cord compression. This assumption is supported by her severe back pain, unilateral weakness, hyperactive reflexes, and sensory changes. The next most appropriate step in her management would be to conduct an MRI of the thoracic spine (option D). This imaging study would provide a detailed view of the spinal cord and surrounding structures, potentially revealing any compressive lesions or metastases that could be causing her symptoms. Other interventions or imaging studies may be considered later, depending on the findings of the MRI.
